# Deep learning methods to predict amyotrophic lateral sclerosis disease progression

**DOI:** 10.1038/s41598-022-17805-9

**Published:** 2022-08-12

**Authors:** Corrado Pancotti, Giovanni Birolo, Cesare Rollo, Tiziana Sanavia, Barbara Di Camillo, Umberto Manera, Adriano Chiò, Piero Fariselli

**Affiliations:** 1 Department of Medical Sciences, University of Turin, 10126 Turin, Italy; 2 Department of Information Engineering, University of Padua, 35131 Padua, Italy; 3 ALS Center, “Rita Levi Montalcini” Department of Neuroscience, University of Turin, 10126 Turin, Italy

**Keywords:** Neurological disorders, Machine learning, Predictive medicine

## Abstract

Amyotrophic lateral sclerosis (ALS) is a highly complex and heterogeneous neurodegenerative disease that affects motor neurons. Since life expectancy is relatively low, it is essential to promptly understand the course of the disease to better target the patient’s treatment. Predictive models for disease progression are thus of great interest. One of the most extensive and well-studied open-access data resources for ALS is the Pooled Resource Open-Access ALS Clinical Trials (PRO-ACT) repository. In 2015, the DREAM-Phil Bowen ALS Prediction Prize4Life Challenge was held on PRO-ACT data, where competitors were asked to develop machine learning algorithms to predict disease progression measured through the slope of the ALSFRS score between 3 and 12 months. However, although it has already been successfully applied in several studies on ALS patients, to the best of our knowledge deep learning approaches still remain unexplored on the ALSFRS slope prediction in PRO-ACT cohort. Here, we investigate how deep learning models perform in predicting ALS progression using the PRO-ACT data. We developed three models based on different architectures that showed comparable or better performance with respect to the state-of-the-art models, thus representing a valid alternative to predict ALS disease progression.

## Introduction

Amyotrophic lateral sclerosis (ALS) is a progressive neurodegenerative disease of adulthood caused by the loss of spinal, bulbar and cortical motor neurons, leading to paralysis of voluntary muscles^1–4^. The actual causes of the disease are still unknown and available treatments aim at extending life expectancy and alleviating symptoms^5–9^. ALS has a considerable variability in progression and outcome, making difficult satisfactory predictions^10,11,11–17^.

For assessing the ALS progression, the most widely used functional scale is the ALS functional rating scale (ALSFRS) or its revised form ALSFRS-R^18–20^. The ALSFRS is a questionnaire that is normally administered to the patients multiple times during the disease progression and it addresses motor skills, such as walking and climbing stairs, breathing, speaking, swallowing, and autonomy in dressing.

In recent years, advanced statistical methods and machine learning have been applied with some success in several ALS studies, e.g. the development of prognostic models for the identification of different risk groups, the patients' stratification through unsupervised methods and the prediction of the ALS evolution over time and of functional impairment through the use of Dynamic Bayesian Networks (DBNs)^21–24^.

Most of the methods have been tested using PRO-ACT data. Although this repository does not perfectly collect a general distribution of the ALS patients in the population, it provides the largest publicly available dataset of merged ALS clinical trials^25^. It has longitudinal data for more than ten thousand patients, including multiple spirometry tests and ALSFRS questionnaires per patient. In 2015, the DREAM-Phil Bowen ALS Prediction Prize4Life Challenge was held on the PRO-ACT data^26,27^, where competitors aimed to predict some disease outcomes such as the slope of the total ALSFRS score between 3 and 12 months and the survival time of patients.

Several machine learning algorithms were developed for the challenge, ranging from linear regression and support vector machines to random forests and Bayesian decision trees^28–30^.This latter approach turned out to be the best performer in the slope prediction. After the challenge, more patients were collected and more models were developed^31^. Given the number of predictive models developed using the PRO-ACT data during the years, it remains an important benchmark dataset for developing new models and comparing them to past results, even though it is known that PRO-ACT patient population has some biases that make it less than ideal for real world applications.

Deep Learning has been successfully applied in several studies on ALS patients, using clinical information with genomic and imaging (MRI) data^32–35^. However, to the best of our knowledge, this is the first time that deep learning approaches are applied to ALS disease progression using the PRO-ACT data for the prediction of the ALSFRS decline. To test the feasibility of using deep learning methodologies for predicting ALS progression, we developed three models (feed-forward, convolutional and recurrent) focusing on the slope prediction in ALSFRS between 3 and 12 months and fast/medium-slow decline classification. These models proved to perform better than those found in the literature in terms of root mean squared error (RMSD) and showed comparable performance in terms of Pearson correlation coefficient.

## Methods

### Datasets description

Data used in the preparation of this study were obtained from the Pooled Resource Open-Access ALS Clinical Trials (PRO-ACT) repository, downloaded on December 10, 2021 from the PRO-ACT website https://ncri1.partners.org/ProACT. The PRO-ACT dataset includes more than 10,000 patients involved in 23 clinical trials and it is split into 13 tables containing information such as time and site of disease onset, ALSFRS questionnaire, demographics, laboratory and treatment data.

These tables were first preprocessed and cleaned to be used in the predictive models. The following tables were considered in the analyses:

- **ALSFRS**: ten questions about the patient ability in everyday motor skills (speaking, walking, swallowing, breathing and others). Answers are integers on a scale from 4 to 0, where 4 is normal and 0 is no ability. Their sum is the total ALSFRS score, used to track the global progression of the disease. Since 1999, a revised questionnaire named ALSFRS-R was introduced^36^ replacing question 10 (breathing) with three more specific questions, 10a (dyspnea), 10b (orthopnea) and 10c (respiratory insufficiency), also on breathing. Since in PRO-ACT we can find both versions, we first followed the DREAM Challenge guidelines and converted the revised version to the original one by taking the value of the revised question 10a as the value of the original question 10 and discarding questions 10b and 10c. According to the same guidelines, we then merged the values of the alternative questions 5a (cutting without gastrostomy) and 5b (cutting with gastrostomy). As the number of patients with ALSFRS-R available is small, the present study was mainly based on the features from the original ALSFRS. However, for the sake of completeness, we also performed predictive analyses on the subset of patients with the additional features from the revised ALSFRS-R.
- **Forced vital capacity**: the maximum amount of air that the patient can exhale after taking the deepest breath possible. The spirometry test for FVC was repeated three times and the maximum value (in liters) was used.
- **Vital signs**: table containing information regarding patients' height and dynamics follow up data such as Systolic and Diastolic Blood Pressure, Pulse, Respiratory Rate and Weight.
- **Riluzole, demographics and onset**: three tables containing information regarding the Riluzole treatment (a drug that can delay respiratory insufficiency and death by some months), age, sex and race, time and site of onset for each patient, respectively.
- **Laboratory**: table containing several laboratory analysis such as blood and urine tests, repeatedly performed at different times during the progression of the disease.

Among these tables, ALSFRS, FVC, Vital Signs and Laboratory are longitudinal, that is, the administration/test time is available, in days. According to the DREAM challenge, we used the time of the first available ALSFRS administration as the reference time for the patient progression and we retained only patients with enough ALSFRS data: at least one ALSFRS administration in the first 3 months (to provide more input data to the predictive model) and at least one ALSFRS after 12 months (to estimate the model outcomes).

As in the challenge, we aimed to predict the progression from the fourth to the twelfth month, using data from the first 3 months. Specifically, the objective was to predict the decline of the total ALSFRS score (the sum of the ten scores) from the fourth to the twelfth month, calculated as the following slope:

$$\begin{aligned} slope=\frac{ALSFRS(t_{2})-ALSFRS(t_{1})}{t_{2}-t_{1}} \end{aligned}$$

were $t_1$ and $t_2$ are the times (in months) of the first visit after 3 and 12 months, respectively.

### Preprocessing

Firstly, we cleaned each table separately, fixing inconsistencies in units of measurements and removing duplicated records.

Then, we created a static summary of the longitudinal data from the first 3 months to be used as features in our models. For each longitudinal feature, we built a vector of seven static summary scores using the available values of the longitudinal feature collected in the first 3 months. The first six scores were computed through summary functions: minimum, maximum, median, standard deviation, first and last observations. The seventh score was the slope between the first and the last observations (when only one observation was available we set the slope as missing).

Features with more than $30\%$ of missing values were discarded and the remaining values were imputed.

To avoid overfitting due to high dimensionality issues, a feature selection via cross validation was performed. In particular, the feature selection consisted in two steps: a feature ranking method was used to sort the variables based on their informativeness for predicting the slope (on the training set) and then the optimal number of features was considered as hyper-parameter and chosen by the network during the optimization process via cross validation. Two feature ranking methods were tested: the impurity-based feature importance from a Random Forest Regressor and the Pearson correlation coefficient.

All these preprocessing steps were performed in Python (version 3.8.3) with the scikit-learn package^37^ (version 0.24.2).

### Assessment and evaluation metrics

To assess the performance of regression models in predicting the ALSFRS slope we used the root mean squared deviation (RMSD)

1 $$\begin{aligned} {\text {RMSD}} =\sqrt{\frac{\sum _{i=1}^{N}(slope^{pred}-slope^{real})^2}{N}}, \end{aligned}$$

and the Pearson correlation coefficient (PCC)

2 $$\begin{aligned} {\text {PCC}} = \frac{Cov(slope^{pred},slope^{real})}{\sigma _{pred}\sigma _{real}}, \end{aligned}$$

where *Cov* is the covariance and $\sigma$ is the standard deviation.

### Hyper-parameters optimization and model evaluation

To avoid biases in the model evaluation and the hyper-parameter selection, we kept $80\%$ of the available individuals into a training set and held out the remaining individuals as test set. On the training set we performed a 5-fold cross-validation to optimize the models' hyper-parameters. To avoid data leakage, imputation, scaling, feature selection and network training were performed independently in each fold. In addition to the networks, each step had its own hyper-parameters which were also optimized via cross-validation. For the imputation, we evaluated both median and mean for continuous values (the mode was fixed for discrete values), while min-max and standardization were considered for scaling. Also the number of features to retain after the feature ranking was optimized. Models were compared by the average PCC on the holdout folds.

Supplementary Tables S1–S4 show the optimal hyper-parameters for each architecture in different tasks. After the hyper-parameters were selected, we retrained the optimal models on the whole training set and estimated their performance on the test set with the appropriate metrics using 10,000 bootstraps to calculate the confidence intervals.

### Deep Learning models

We tested three different deep learning architectures. The first is a standard feed-forward neural network (FFNN) that takes as input the selected static and summarized longitudinal features. It has three hidden layers with dropout regularization. We used a linear activation function for the output layer and the mean squared error as loss function.

**Figure 1:**
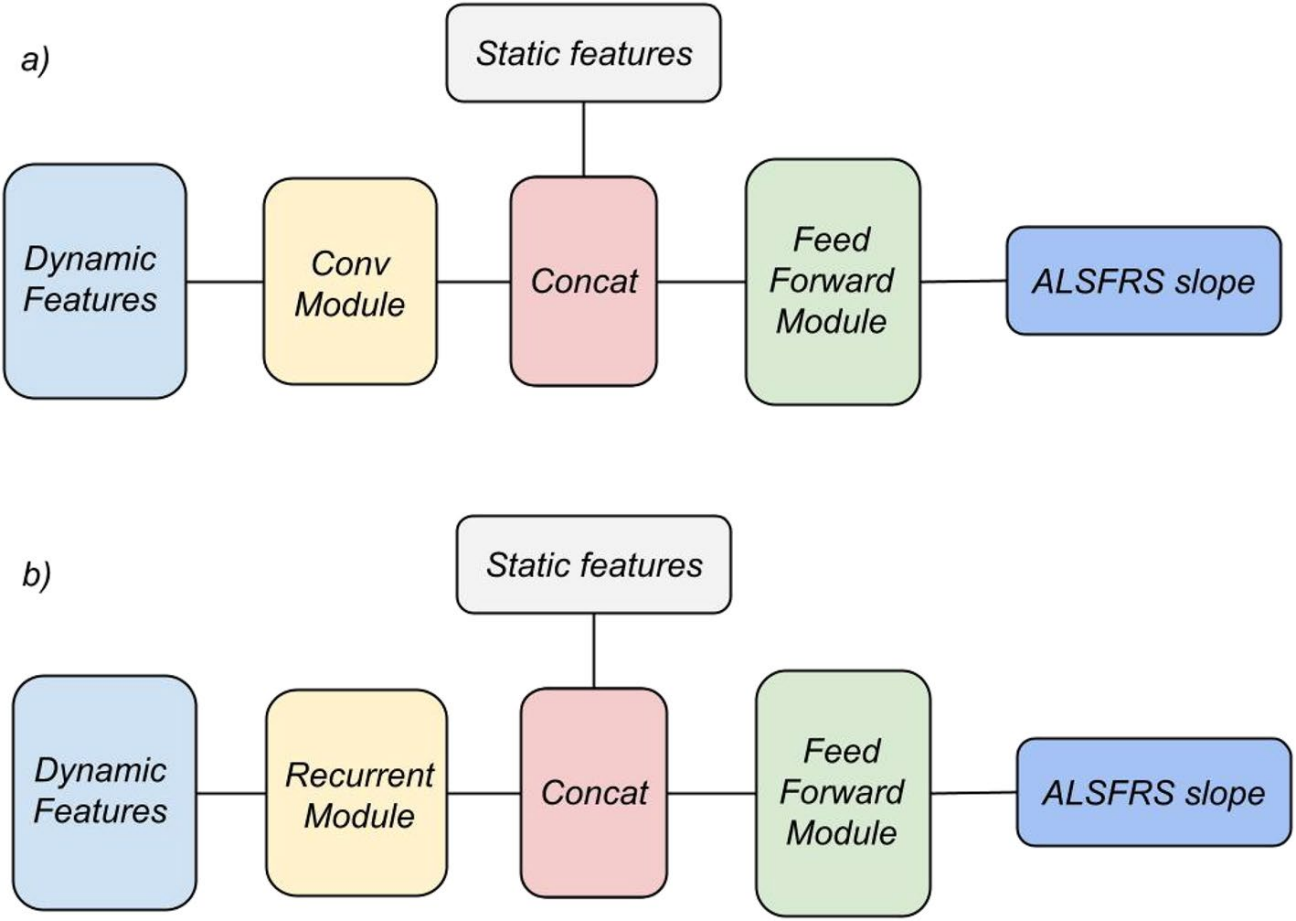
(**a**) Convolutional neural network architecture. The dynamic features of the questionnaire dataset flows into the convolutional module constituted by two layers; after the concatenation with the static features, the information is fed into a feed-forward neural network to predict the two outcomes. (**b**) Recurrent Neural Network architecture. The structure is the same as the convolutional architecture except for the Recurrent module that processes the dynamic features.

The second architecture is a convolutional neural network that can handle longitudinal data directly. Its input was divided into two parts: the longitudinal ALSFRS data from the first 3 months and the residual previously-selected static and summarized longitudinal features (without the ALRFRS-derived features). Since in the ALSFRS questionnaire dataset the maximum number of visits in the first 3 months was 5, we constructed for each patient a matrix $M\in R^{n\times m}$ where $n=11$ and $m=5$. Specifically, *n* is the number of ALSFRS-related questions with the time they were collected; *m* is the maximum number of visits. For those patients with less than 5 visits, we replaced the missing data with the value of the last visit. Hence, the information passes through two convolutional layers, whose number of filters was chosen during the optimization process; each filter of the first layer, which performs the convolution operation on the input matrix, was taken with a size of $11\times 3$. The first filter dimension represents the number of ALSFRS questions considered (and the time they are measured), while the second one represents the width of the time window. Consequently, each filter of the second layer (with size $1\times 3$) performs the same operation and the extracted features are concatenated with the other static features and fed into a feed-forward neural network (with the same architecture as the FFNN). A diagram is available in Fig. 1.

The third architecture is a standard recurrent neural network (RNN), which was purposely built to handle longitudinal data, similarly to what done for the CNN. The questionnaire information $M\in R^{n\times m}$ is passed to two Recurrent layers, whose number of nodes was chosen during the optimization process. The extracted features are then concatenated with the other static features and fed into a feed-forward neural network (again with the same architecture as the FFNN), as displayed in Fig. 1.

The optimal network hyper-parameters (hidden layer sizes, activation function, dropout rate ) and training parameters (batch size, epochs) were determined independently for each architecture via cross-validation on the training set. All the networks were implemented using TensorFlow (version 2.5.0).

### Survival

Date of death is available for a subset of patients in PRO-ACT. Other patients were treated as censored at the last available ALSFRS administration. Survival results were produced using the Kaplan–Meier estimators and the log-rank test implemented in the Python package lifelines (version 0.26.4).

## Results

### Data

After removing patients without enough ALSFRS/ALSFRS-R administrations and features with too many missing values (see methods), the main processed dataset considered for the analyses includes 2921 patients and 274 features. Applying the same filters to the patients with available ALSFRS-R data, we obtained a smaller dataset of 1473 patients and 204 features. A brief description of the main cohort is shown in Table 1, while those for the patients with ALSFRS-R and for the patients that were excluded for the main cohort are in Supplementary Tables S5 and S6, respectively.

**Table 1:** ALSFRS/ALSFRS-R cohort descriptive statistics. For each variable, the number and percentage of non missing values within the dataset before imputation, as well as and their percentage or median values are reported. Total ALSFRS and weight refer to the first available observation.

Data	Count (observed rate)	Percentage/median (iqr)
Age	2581 (88.4%)	55 (46–63) years
Sex	2921 (100%)	63.4% males
Height	2577 (88.2%)	171 (164–178) cm
Caucasian	2870 (98.2%)	95.5%
Weight (first)	2715 (92.9%)	76.0 (65.0–86.4)
Time of onset	2864 (98%)	558 (367–819) days
Spinal; Bulbar; Both Spinal and Bulbar; Others	2534 (86.7%)	67.8%; 20.8%; 0.7% 10.7%
Total ALSFRS (first)	2921 (100%)	32 (28–35)
Riluzole use	2505 (85.7%)	73.7% yes

**Figure 2:**
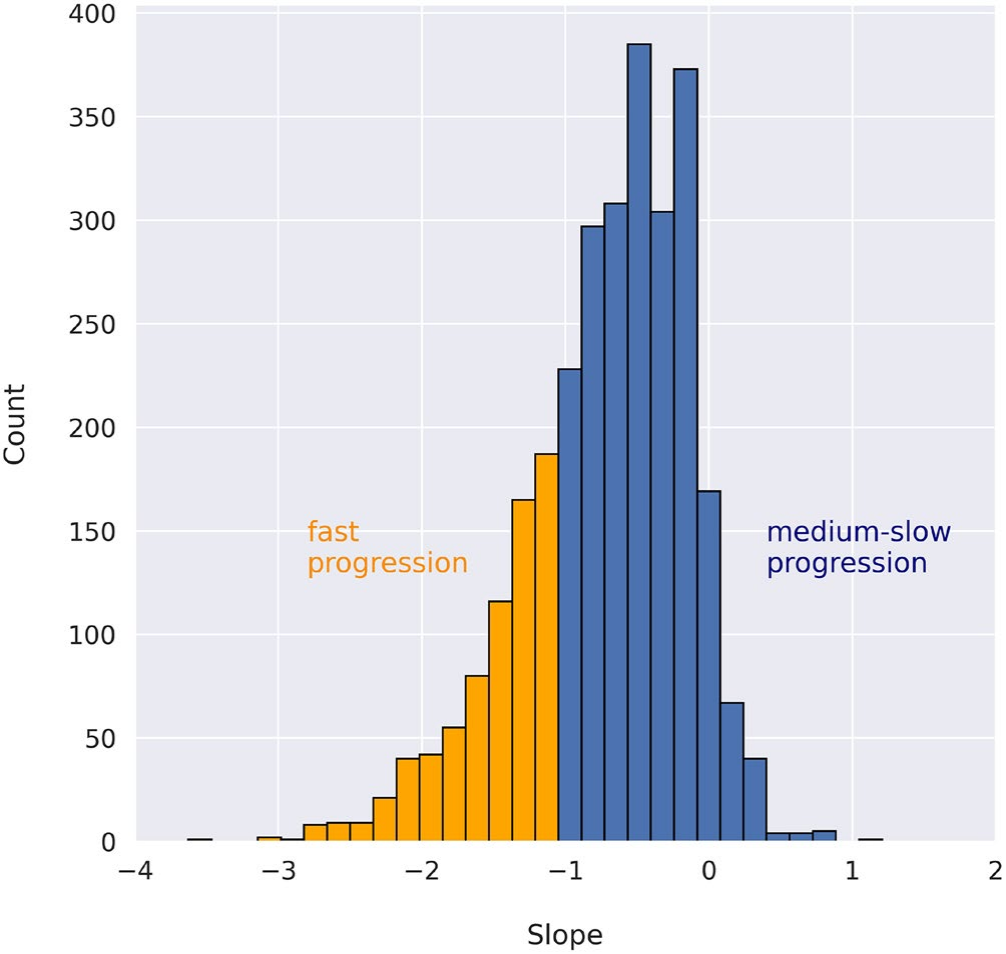
Distribution of the 3–12 months ALSFRS slope distribution and fast versus medium-slow progressors.

The main outcome of the developed models was to predict the slope of the total ALSFRS score between 3 and 12 months, whose distribution is shown in Fig. 2.

### Feature Ranking and Selection

When developing a machine learning method, it is important to consider the problem of dimensionality that can often lead to overfitting, especially when the dataset is not sufficiently large. Hence, in order to reduce the dimensionality and to select the most relevant features, we tested two ranking methods on the training set, the Random Forest feature importance and the Pearson correlation coefficient. Subsequently, the optimal number of the top-ranked features was selected during the optimization process for the FFNN and used for all the other models. The FFNN optimized with the top 30 Random Forest ranked features had a better cross-validation performance than with the correlation ranked ones, as shown in Supplementary Table S7.

**Figure 3:**
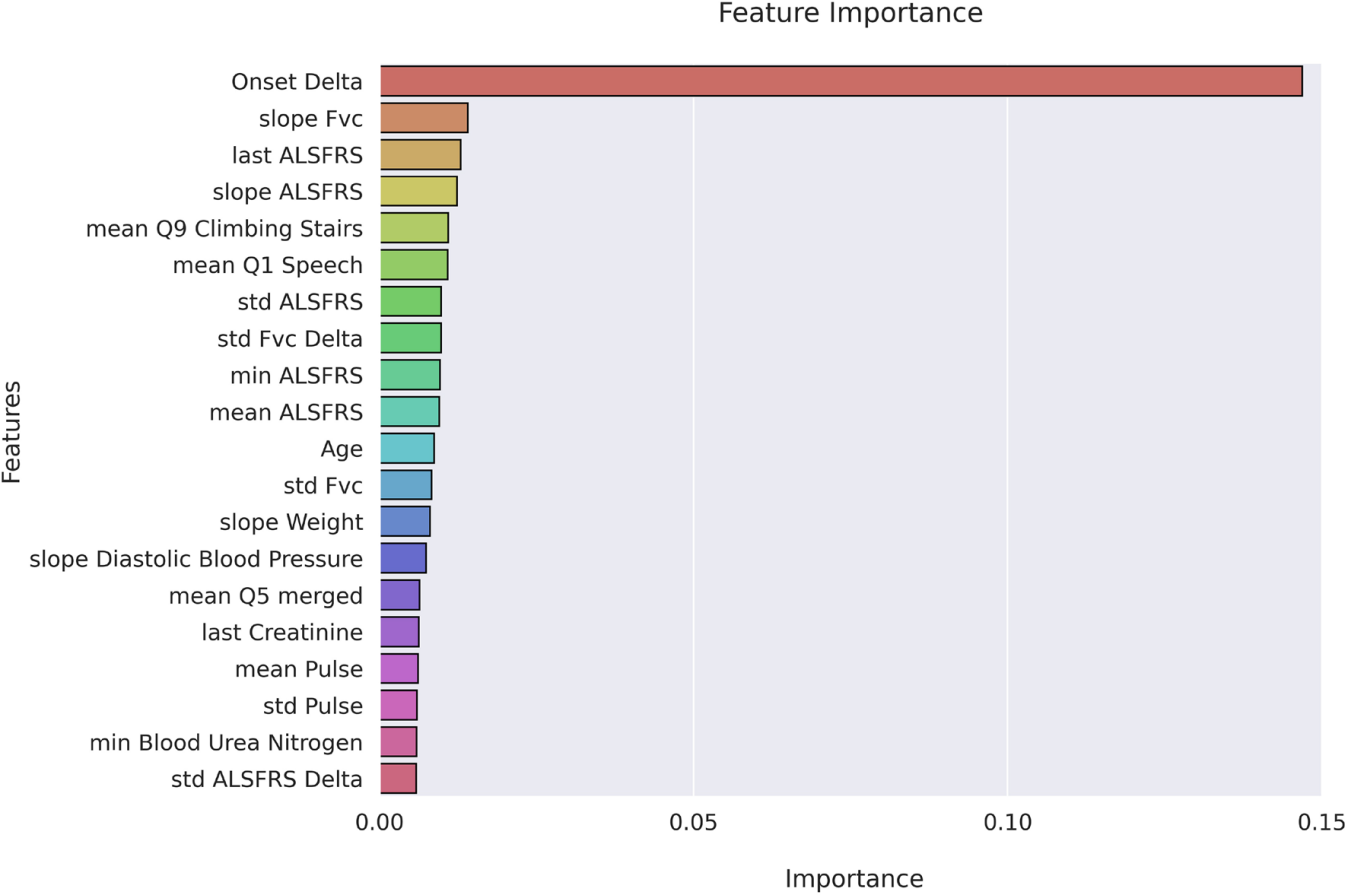
Feature importance. Top 20 most important features ranked by Random Forest via cross-validation on the training set, using the Gini criterion.

Fig. 3 shows the top 20 features ranked by the Random Forest and in Supplementary Fig. S1 those ranked by the correlation-based method.

All the ranking methods agree that the “Onset Delta” is the most predictive feature, followed by FVC, ALSFRS, Age, Weight, and others.

### ALSFRS slope prediction

In the original 2015 DREAM Phil Bowen ALS Challenge, solvers had just 918 patients for training their models, which were then evaluated on an external validation set, available only to the organizers. Since then the PRO-ACT repository grew, allowing us to have 2921 patients. To avoid an unfair comparison between ours and the challenge models, we selected two state-of-the-art models^31^ as reference, which use a similar number of individuals (n = 2454) extracted from the PRO-ACT repository to predict the ALSFRS slope. The models are a Random Forest Regressor (RF) and a Bayesian Additive Regression Trees (BART), and this latter has shown slightly better performance.

The performance of our and the reference models is shown in Table 2 in terms of Root Mean Squared Deviation (RMSD) and Pearson’s Correlation Coefficient (PCC). The scores are very close and the confidence intervals are overlapping, indicating similar performance among the models. The best performing architecture seems to be the FFNN+CNN. Our models have a slightly lower error than RF and BART, but a slightly lower correlation than BART. In Supplementary Table S8 we also reported the running time for the FFNN training compared to a Linear Regression and a Random Forest with 100 trees.

A smaller subset of about half of the patients had information on the more recent and accurate ALSFRS-R scale. Running the models on this subset and using the ALSFRS-R slope resulted in lower scores, as reported in Table S9. However, the performance drop may be due to different factors, including the reduced amount of data provided, making any speculations unsupported.

**Table 2:** ALSFRS slope prediction performance. RMSD and PCC are shown for all methods. FFNN, CNN, and RNN performance was obtained on the external test set (n = 731) using 10,000 bootstrap with resampling. For these predictors we reported the mean and the 95% confidence interval (CI). FFNN+CNN represents the ensemble prediction of the two neural networks. The best values for each metric are highlighted in bold. *Random Forest (RF) and Bayesian Additive Regression Trees (BART) are taken from literature^31^, where they are reported without CI.

Methods	RMSD	PCC
FFNN	0.528 (0.502–0.555)	0.451 (0.404–0.495)
CNN	0.527 (0.499–0.556)	0.439 (0.388–0.487)
RNN	0.529 (0.501–0.558)	0.429 (0.379–0.476)
FFNN+CNN	**0.521** **(0.494–0.548)**	0.462 (0.415–0.508)
RF*	0.563	0.446
BART*	0.554	**0.472**

**Figure 4:**
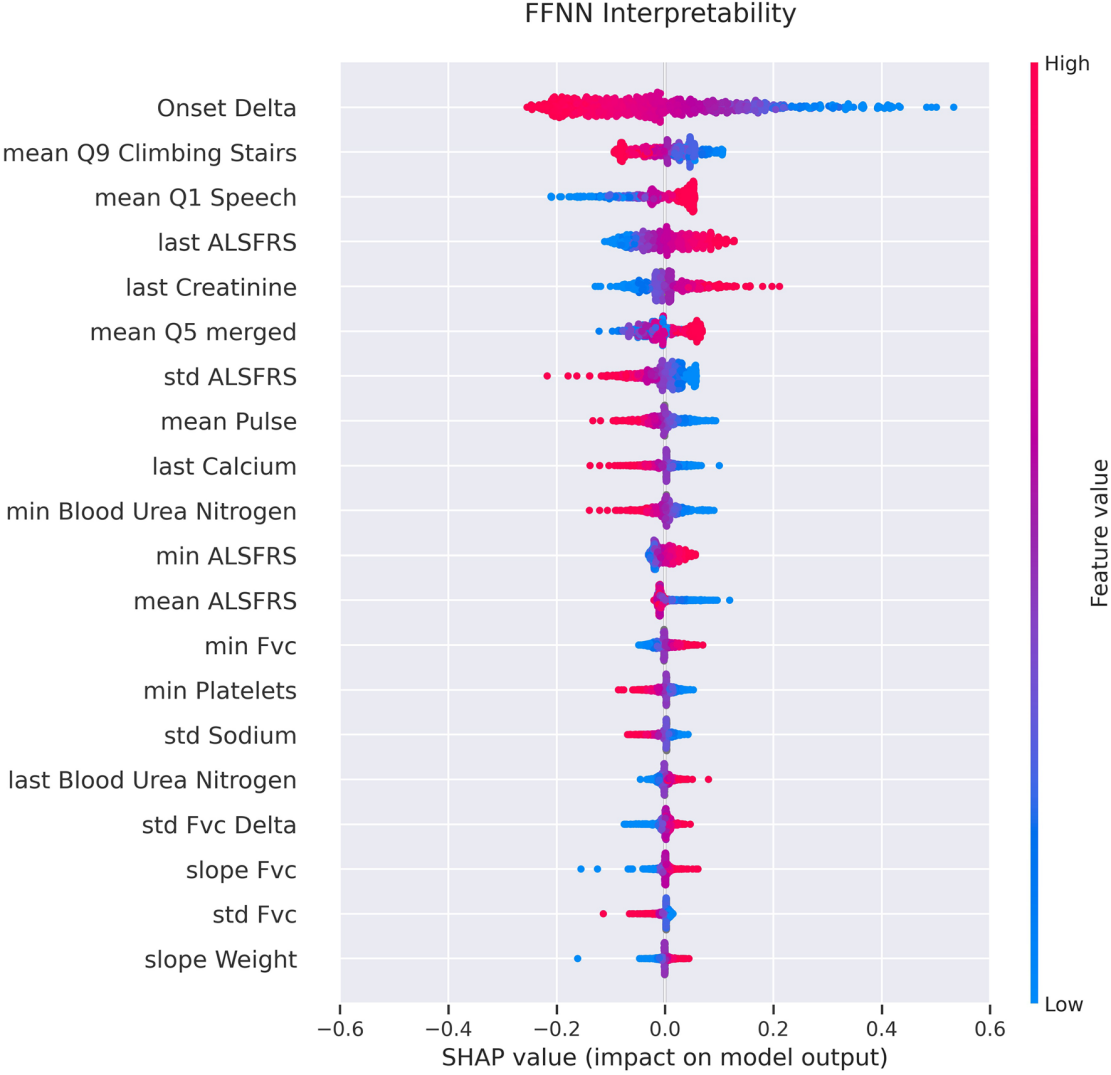
Shapley values for the FFNN architecture; x-axis: the impact on the model output, y-axis: the top 20 most predictive features. The colormap represents the feature values.

In order to improve interpretability, we created a beeswarm plot (Fig. 4) using the SHAP python library^38^ for the FFNN network. Shapley values represent the impact of having a certain value for a given feature, in comparison to the prediction obtained if that feature took a baseline value. This graph shows to which extent and how each variable impacts on the output of the model, providing important insights into the interpretability of the model.

We also investigated how the performance varies when a reduced number of features is considered. Specifically, we trained again the FFNN using only the top five features (Onset Delta,slope FVC, last ALSFRS, slope ALSFRS, and mean Q9 climbing). CNN and RNN were also retrained using the entire ALSFRS questionnaire (longitudinal data) and the features Onset Delta, slope FVC, last ALSFRS and slope ALSFRS. From Table 3 it can be seen that, although there is a drop for all the three architectures, the performance of the FFNN and especially that one of the CNN remains rather stable. On the other hand, the RNN correlation drops more noticeably.

**Table 3:** ALSFRS slope performance with most important features. RMSD and PCC are shown for all methods. Performance was obtained on the external test set (n = 731) using 10,000 bootstrap with resampling. FFNN was trained using the top 5 features, CNN and RNN were trained using ALSFRS questionnaire data and the top 4 features. The best values for each metrics are in bold.

Methods	RMSD	PCC
FFNN	0.534 (0.506–0.564)	0.414 (0.367–0.458)
CNN	**0.530(0.501–0.559)**	**0.429(0.379–0.476)**
RNN	0.544 (0.514–0.574)	0.375 (0.328–0.419)

### Role of imputation

We then explored how imputation could influence and bias our predictions. In particular, we compared two FFNN models using the top 5 features, one trained and optimized on the imputed dataset (n = 2338) and the other on the non-imputed dataset (n = 1748). Both models were finally tested on the same non-imputed test set (n = 583). Even if the models were independently optimized, the results were basically the same in terms of RMSD and PCC, as shown in Table 4, suggesting that the imputation has no particular effect on the model outcome. However, it is worth noticing that to avoid any source of bias, for all the models considered in this study the imputation was performed using only the training data information for both training and the corresponding test sets. This procedure is crucial since a single imputation based on the whole dataset would lead to over-optimistic results and it must be avoided.

**Table 4:** Assessing the impact of the missing values imputation on ALSFRS slope performance with the top 5 most important features. RMSD and PCC are presented for the two FFNN methods: FFNN was trained on imputed n = 2338 patients, while FFNN$^*$ on n = 1748 non-imputed ones. Both models were then tested on n = 583 non-imputed examples.

Methods	RMSD	PCC
FFNN	0.547 (0.512–0.582)	0.416 (0.359–0.472)
FFNN$^*$	0.546 (0.511–0.581)	0.415 (0.354–0.475)

### Survival

**Figure 5:**
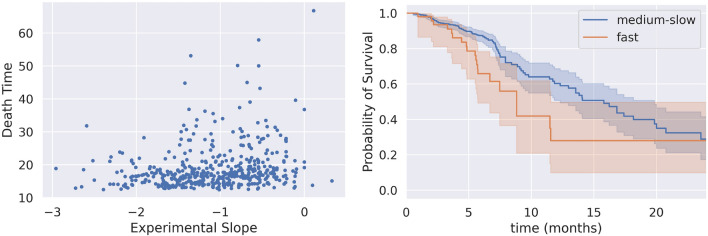
Slope and survival. Left: Scatterplot of the experimental slope between months 3 and 12 against the time-to-death of 458 patients. Right: Kaplan–Meier curves for fast and medium-slow progressing patients in the test set. Fast and medium-slow progressors were 21 and 105, respectively. Times start from month 12 after the first visit.

We also explored the relation between ALSFRS slope and survival, taking advantage of the availability of the time-to-death for 458 patients in our dataset. The experimental slope shows a weak PCC of 0.2 with the time-to-death (Fig. 5, left panel). Nevertheless, the predicted ALSFRS slope can give some insight in the survival of patients. Patients were split according to their predicted slope by the FFNN model into two groups, fast and medium-slow progressors, with a threshold of $-1.1$^26^. The fast progressors showed a significantly shorter survival in both the training and test sets (log-rank test p-value < 0.005).

## Discussion

In this study, we used the widely adopted PRO-ACT dataset. It is worth noticing that this dataset is not representative of a general ALS distribution. The patients are younger than average, with disease duration from onset of almost 2 years, average first ALSFRS of 31, and prevalence of spinal onset. Nonetheless, PRO-ACT allowed us to compare our results with those from existing methods not based on deep learning approaches that were trained on the same data. We tested three deep-learning architectures, two of which combining static and longitudinal data.

While deep learning models performed comparably to state-of-the-art models, they did not provide a decisive advantage, especially considering wide confidence intervals and some differences in the dataset and the testing methodology with respect to the available literature models.

Deep learning models seem to have a slight advantage in terms of RMSD but a slight disadvantage in terms of PCC. The same holds for the different architectures, even though the CNN+FFNN model seems to slightly outperform the others. However, no architecture holds a clear advantage with respect to the other models. While future models can always overcome the previous ones, the close performance of our models and those from the literature suggest a possible upper limit to the achievable precision of the ALSFRS slope prediction.

Deep learning models are considered hard to explain and interpret. However, we can see that many features contribute to the slope regression using Shapley values. While the time from ALS onset to the diagnosis still plays a prominent role, as for the Random Forest model, many other features impact the slope prediction. Unsurprisingly, many of these are directly related to the ALSFRS questionnaire, but others are not, like creatinine or FVC. Despite the limits observed in the model performance, we showed that the predicted ALSFRS slope is able to distinguish fast progressors with worse survival from medium-slow progressors and is thus predictive of concrete clinical events in the patient history.

## Supplementary Information

Supplementary Information.

## Data Availability

All data used in this study were obtained from the Pooled Resource Open-Access ALS Clinical Trials (PRO-ACT) repository. The dataset is provided by the PRO-ACT Consortium members and it is easily accessible after registration at the PRO-ACT website https://ncri1.partners.org/ProACT.
