# Supplementary material for: Deep learning methods to predict amyotrophic lateral sclerosis disease progression
Source: Sci Rep. 2022 Aug 12;12:13738. doi: 10.1038/s41598-022-17805-9 (PMC9374680; doi:10.1038/s41598-022-17805-9)
Supplement: Supplementary file 1 — Supplementary Information. [file 41598_2022_17805_MOESM1_ESM.pdf]

S1 Supplementary Materials for "Deep Learning methods to predict Amyotrophic Lateral Sclerosis disease progression"

Corrado Pancotti, Giovanni Birolo, Cesare Rollo, Tiziana Sanavia, Barbara Di Camillo Umberto Manera, Adriano Chiò, Piero Fariselli

S1.1 Optimized Architectures

The following tables contain the best hyperparameters obtained during the optimization process in 5-fold cross validation.

Table S1: Optimized architectures ALSFRS. Best hyperparameters for FFNN, CNN and RNN, obtained via 5-fold cross validation for ALSFRS slope prediction

**Parameters**	**FFNN**	**CNN**	**RNN**
Convolutional module (activation)	-	16-16 (sigmoid-sigmoid)	-
Recurrent module (activation)	-	-	16-8 (relu-relu)
Hidden units	64-16-8	32-32-8	64-32-8
Activation	relu-tanh-tanh	sigmoid-tanh-tanh	relu-relu-relu
Dropout	0.1-0.1-0.05	0.1-0.0-0.05	0.1-0.05-0.00
Epochs	200	50	50
Batch-size	32	32	32
Optimizer	RMSProp	Adam	Adam
Loss	Mse	Mse	Mse
Scaler	MinMax	Standard	MinMax

S1.2 Descriptive statistics

The two following tables contains descriptive statistics for the PRO-ACT cohort with ALSFRS-R available (n=1473) after the cleaning process and also for the discarded PRO-ACT population (n=7821) not included in this study.

S1.3 Feature ranking

In the following figure we showed the top 20 correlated features with the ALS-FRS slope and in the table a comparison of the performance in cross validation for two FFNN, one optimized with top 30 features ranked by the correlation based method (FFNN-Corr) and the other by the Random Forest Regressor using the Gini criterion (FFNN-Gini).

Table S2: Optimized architectures ALSFRS-R. Best hyperparameters for FFNN, CNN and RNN, obtained via 5-fold cross validation for ALSFRS-R slope prediction

**Parameters**	**FFNN**	**CNN**	**RNN**
Convolutional module (activation)	-	16-16 (sigmoid-sigmoid)	-
Recurrent module (activation)	-	-	16-16 (relu-relu)
Hidden units	64-16-8	32-8-32	32-8-32
Activation	relu-relu-relu	sigmoid-tanh-tanh	tanh-tanh-tanh
Dropout	0.1-0.1-0.05	0.1-0.1-0.00	0.1-0.05-0.00
Epochs	200	50	150
Batch-size	64	32	64
Optimizer	Adam	RMSProp	RmsProp
Loss	Logcosh	Mse	Mse
Scaler	MinMax	Standard	MinMax

Table S3: Optimized architectures ALSFRS top 5. Best hyperparameters for FFNN, CNN and RNN, obtained via 5-fold cross validation for ALSFRS slope with top 5 features prediction

**Parameters**	**FFNN**	**CNN**	**RNN**
Convolutional module (activation)	-	48-16 (sigmoid-sigmoid)	-
Recurrent module (activation)	-	-	32-8 (relu-relu)
Hidden units	16-8-8	64-16-32	32-8-8
Activation	sigmid-sigmoid-sigmoid	sigmoid-sigmoid-sigmoidh	sigmoid-tanh-tanh
Dropout	0.1-0.05-0.00	0.1-0.00-0.05	0.05-0.00-0.1
Epochs	200	200	150
Batch-size	128	128	64
Optimizer	Adam	RMSProp	Adam
Loss	Mse	Mse	Mse
Scaler	StandardScaler	StandardScaler	MinMax

S1.4 Running Time comparison

S1.5 ALSFRS-R slope prediction

In the following table we reported the prediction of ALSFRS-R slope of the three neural network architectures presented in this study.

Table S4: Optimized architectures ALSFRS Missing. Best hyperparameters for FFNN and FFNN*, obtained via 5-fold cross validation for ALSFRS slope. FFNN uses (n=2338) imputed examples for the training, while FFNN* (n=178) non imputed examples.

**Parameters**	**FFNN**	**FFNN***
Hidden units	64-8-8	64-32-64
Activation	tanh-sigmoid-sigmoid	relu-relu-relu
Dropout	0.15-0.00-0.00	0.1-0.15-0.05
Epochs	150	100
Batch-size	256	256
Optimizer	Adam	RMSProp
Loss	Mae	Mae
Scaler	StandardScaler	StandardScaler

Table S5: Descriptive statistics for ALSFRS-R cohort (n=1473)

**Data**	**Count (observed rate)**	**Percentage/Median (iqr)**
Age	1133 (76.9 %)	56 years (47 - 64)
Sex	1473 (100 %)	63.1 % males
Height	1125 (76.4 %)	172 cm (165 - 179)
Caucasian	1421 (96.5 %)	94.8 %
Weight (first)	1267 (86.0 %)	77.7 kg (67.4 - 88.6)
Time of onset	1422 (96.5 %)	526 days (350 - 694)
Spinal;Bulbar;Both;;Others	1163 (79.0 %)	55.5%; 20.2%; 0.7% 23.6%
Total ALSFRS-R (first)	1473 (100.0 %)	39 (35 - 43)
Riluzole use	1128 (76.6 %)	86.0 % yes

Table S6: Descriptive statistics for the discarded cohort (n=7821)

**Data**	**Count (observed rate)**	**Percentage/Median (iqr)**
Age	5121 (65.6 %)	59 (49 - 66) years
Sex	7796 (99.9 %)	59.1 % males
Height	4730 (60.6 %)	169 (162 - 176) cm
Caucasian	4185 (53.6 %)	95.0 %
Weight (first)	4829 (61.9 %)	72.0 (62.0 - 82.5) kg
Time of onset	4013 (51.4 %)	541 (359 - 828) days
Spinal;Bulbar;Both;;Others	2244 (28.7 %)	74.0%; 21.5%; 0.8% 3.7%
Total ALSFRS (first)	3843 (49.3 %)	29 (24 - 33)
Riluzole use	6312 (80.9 %)	80.9 % yes

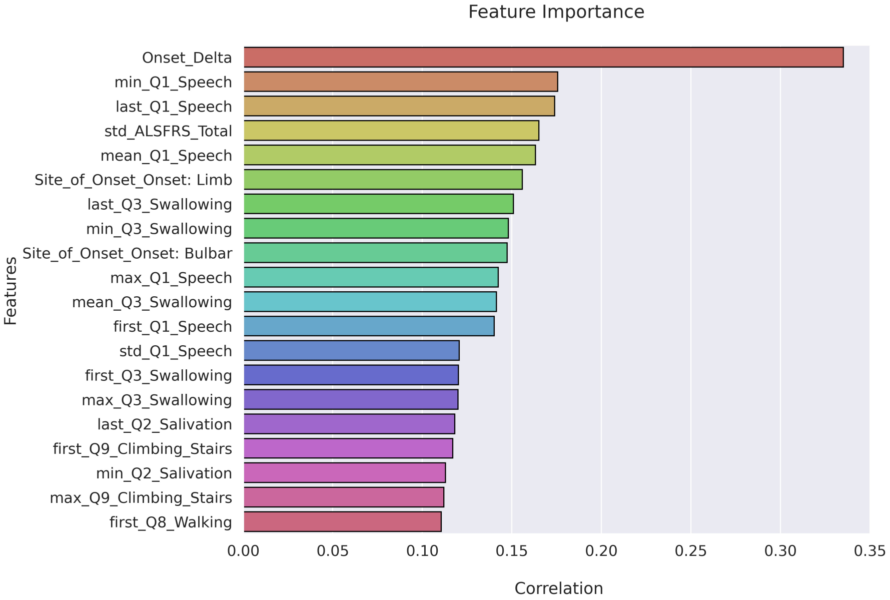

Fig. S1: 20 most Correlated features with ALSFRS slope

Table S7: ALSFRS slope comparison performance. RMSD and PCC for the FFNN-Corr and for the FFNN-Gini. Performance are calculated in a 5 fold cross validation

**Methods**	**RMSD**	**PCC**
FFNN-Corr	0.533 ± 0.02	0.414 ± 0.03
FFNN-Gini	0.536 ± 0.03	0.428 ± 0.03

Table S8: Running Time comparison. Running time comparison for the optimized Feed Forward Neural Network (FFNN), a Random Forest (RF) with 100 estimators, and a Logistic Regression (LR)

**Methods**	**CPU time**	**Wall Time**
FFNN	73.0 s	37.1 s
RF	23.5 ms	10.6 ms
LR	3.6 s	3.32 s

Table S9: ALSFRS-R slope performance. As before RMSD and PCC are shown for all methods. Performance are obtained on the external test set (n=369) with 10000 bootstrap with resampling

**Methods**	**RMSD**	**PCC**
FFNN	0.666 (0.626-0.707)	0.364 (0.283-0.442)
CNN	0.633 (0.585-0.682)	0.337 (0.257-0.413)
RNN	0.638 (0.588-0.687)	0.324 (0.247-0.398)
